# Knowledge, attitude and perception towards COVID-19 among representative educated sub-Saharan Africans: A cross-sectional study during the exponential phase of the pandemic

**DOI:** 10.1371/journal.pone.0281342

**Published:** 2024-02-01

**Authors:** Aniefiok John Udoakang, Nora Nghochuzie Nganyewo, Alexandra Lindsey Djomkam Zune, Charles Ochieng’ Olwal, Nsikak-Abasi Aniefiok Etim, Mary Aigbiremo Oboh, Kesego Tapela, Francis Dzabeng, Samuel Mawuli Adadey, Agnes Udoh, Mazo Koné, Joe Kimanthi Mutungi, Peter Kojo Quashie, Gordon Akanzuwine Awandare, Lily Paemka

**Affiliations:** 1 West African Centre for Cell Biology of Infectious Pathogens (WACCBIP), College of Basic and Applied Sciences, University of Ghana, Legon, Accra, Ghana; 2 Department of Biosciences and Biotechnology, University of Medical Sciences, Ondo City, Ondo State, Nigeria; 3 Department of Biochemistry, Cell and Molecular Biology, College of Basic and Applied Sciences, University of Ghana, Accra, Ghana; 4 Medical Research Council Unit, The Gambia at the London School of Hygiene and Tropical Medicine, Banjul, The Gambia; 5 Department of Agricultural Economics and Extension, University of Uyo, Uyo, Akwa Ibom State, Nigeria; 6 Jones School of Business, Rice University, Houston, Texas, United States of America; 7 Department of Zoology, University of Ibadan, Ibadan, Oyo State, Nigeria; 8 The Francis Crick Institute, London, United Kingdom; 9 Virology Department, Noguchi Memorial Institute for Medical Research, University of Ghana, Legon, Accra, Ghana; Lagos State University, NIGERIA

## Abstract

Coronavirus disease 2019 (COVID-19) pandemic, caused by the Severe Acute Coronavirus 2 (SARS-CoV-2), is a global health threat with extensive misinformation and conspiracy theories. Therefore, this study investigated the knowledge, attitude and perception of sub-Saharan Africans (SSA) on COVID-19 during the exponential phase of the pandemic. In this cross-sectional survey, self-administered web-based questionnaires were distributed through several online platforms. A total of 1046 respondents from 35 SSA countries completed the survey. The median age was 33 years (18–76 years) and about half (50.5%) of them were males. More than 40% across all socio-demographic categories except the Central African region (21.2%), vocational/secondary education (28.6%), student/unemployed (35.5%), had high COVID-19 knowledge score. Socio-demographic factors and access to information were associated with COVID-19 knowledge. Bivariate analysis revealed that independent variables, including the region of origin, age, gender, education and occupation, were significantly (p *<* 0.05) associated with COVID-19 knowledge. Multivariate analysis showed that residing in East (odds ratio [OR]: 7.9, 95% confidence interval (CI): 4.7–14, p*<*0.001), Southern (OR: 3.7, 95% CI: 2.1–6.5, p*<*0.001) and West (OR: 3.9, 95% CI: 2.9–5.2, p*<*0.001) Africa was associated with high COVID-19 knowledge level. Apart from East Africa (54.7%), willingness for vaccine acceptance across the other SSA regions was <40%. About 52%, across all socio-demographic categories, were undecided. Knowledge level, region of origin, age, gender, marital status and religion were significantly (p < 0.05) associated with COVID-19 vaccine acceptance. About 67.4% were worried about contracting SARS-CoV-2, while 65.9% indicated they would consult a health professional if exposed. More than one-third of the respondents reported that their governments had taken prompt measures to tackle the pandemic. Despite high COVID-19 knowledge in our study population, most participants were still undecided regarding vaccination, which is critical in eliminating the pandemic. Therefore, extensive, accurate, dynamic and timely education in this aspect is of ultimate priority.

## Introduction

In December 2019, a cluster of pneumonia of unknown aetiology was identified in Wuhan, China [1]. The novel severe acute respiratory syndrome coronavirus 2 (SARS-CoV-2) was subsequently identified as the cause [2], and the resultant disease was later termed the coronavirus disease 2019 (COVID-19) [3]. In Africa and sub-Saharan Africa (SSA), respectively, the first cases of COVID-19 were confirmed in Egypt on the 14th and in Nigeria on the 27th February 2020. By 11th March 2020, the disease was declared a pandemic by the World Health Organization (WHO) [4]. As of 29th April 2021, there have been 150,793,624 confirmed cases and 3,171,078 associated deaths, with about 3.0% and 3.8%, respectively, of the cases and associated deaths in Africa [5]. Although only four SSA countries (South Africa, Nigeria, Kenya and Ghana) are among the top ten contributing to the COVID-19 burden on the continent, they bear about 43.6% of the total cases, with South Africa having the highest burden. The COVID-19 pandemic has caused serious health, social and economic challenges, some of which are directly linked to demographic factors.

Although most governments and global health organizations have disseminated useful and accurate COVID-19 information, extensive and intensive infodemics have also accompanied the pandemic, especially across social networking media/sites. This inaccurate information has impacted global and national efforts in combating the COVID-19 pandemic, including vaccines acceptance [6]. The virus has continued to spread despite concerted efforts by governments, and behavioural modifications by citizens, to mitigate its transmission [7], raising concerns associated with increased transmission and immune evasion with the emergence of different SARS-CoV-2 variants [8].

Besides the innate history and natural science of any disease, at-risk populations’ demographic characteristics play a vital role in the type and intensity of interventive measures necessary to curb it [9–11]. Hence, disease knowledge is usually considered the first approach to any implementable health mitigation strategy [12, 13], increasing public awareness of preventive measures to curb transmission. Therefore, the population’s awareness and perception, of a disease outbreak, could prompt positive or negative dispositions [14] that could either augment or stem the disease’s tide.

Although several studies have documented demographic characteristics and the populations’ knowledge, attitude and perception towards COVID-19 [7, 15], there is scant data capturing this in Africa, with only about 3% of COVID-19 publication output from the continent [16, 17]. A KAP study in Egypt and Nigeria showed that participants had sufficient COVID-19 knowledge and a positive attitude, while only about one-fifth of the respondents were satisfied with their government’s management of the disease [15]. In a Kenyan study, high knowledge did not translate to a positive attitude, as 61% of participants felt that COVID-19 measures would negatively impact their livelihood source [18]. Similarly, a North American COVID-19 study showed that knowledge did not translate into practice as less than one-sixth of the study participants wore a face mask [19]. Conversely, high COVID-19 knowledge translated into a positive attitude and practice in Malaysia, although only about half of the respondents wore face masks [20].

Poor COVID-19 knowledge and practices were observed among chronic disease patients in Ethiopia [21]. As part of a comprehensive measure to suppress SARS-COV-2 transmission, the WHO has recommended using a mask, although this alone does not provide adequate protection against the virus [22]. Therefore, being a novel disease, more COVID-19 studies are needed to help draw up timely policies and take necessary measures towards curbing this pandemic and possible future threats. Data from different parts of the sub-region using a single data collection tool will give us a homogenous picture of the true KAP within SSA. Hence, this study was conducted to investigate knowledge, attitude and perception towards COVID-19 among representative educated sub-Saharan Africans.

## Methods

### Study setting and sample size

Sub-Saharan Africa (S1 Table) comprises 48 independent countries partially located south of the Sahara desert [23] with a landscape distribution over an estimated 23.85 million km^2^ [24]. It has an estimated 1 billion population, the third largest region worldwide after Eastern and Southern Asia.

The sample size was calculated using the “Raosoft” sample size calculator [25], with a SSA population size of 1,122,845,156 people [26], 3% error margin, 95% confidence level and a 50% response distribution. A sample size of 1068 was obtained for this study, but we received responses from 1,046 representative educated sub-Saharan Africans.

### Data collection and management

The survey questionnaire consisted of 33 questions divided into four sections: demographics, knowledge, attitude and perception (S2 Table). It was developed in English and translated to French, Portuguese and Spanish (S1 File: A, B, C and D, respectively). There were 13 demographic, 13 knowledge, 2 attitude and 5 perception items. To validate the translation from English to other languages, the translated versions were cross-checked and then back-translated to English to ensure the meaning of the content was maintained. Following a critical review of the survey contents, the questionnaire was pre-tested for acceptability, clarity, comprehension, challenges, and timelines in completing the survey, prior to being applied in the study. The questionnaire required about 7 minutes to complete.

The validated self-administered online questionnaire was disseminated over different platforms, including Email, Telegram, Twitter, WhatsApp, Instagram, and Facebook, through a Uniform Resource Locator (URL) link using Google Docs. The convenience cross-sectional online sampling was done without restriction to geographical location. The target population were educated SSAs worldwide, who were 18 years old and above. The demographic factors were to discover participants’ demographics and KAP towards the COVID-19 pandemic. The knowledge domain was to assess respondents’ SARS-CoV-2 and COVID-19 knowledge, including SARS-CoV-2 mode of transmission and COVID-19 aetiology, preventive measures, and clinical manifestation. The attitude domain included participants responses to the pandemic such as vaccine acceptance, whereas the perception domain related to individual’s opinions and beliefs like their governments’ response regarding the pandemic. While some knowledge questions required selecting the correct answer(s), most required either yes/true or no/false response. Other questions required scoring on a five-point Likert scale (1, strongly disagree; 2, disagree; 3, neutral 4, agree; 5, strongly agree).

We conducted the survey between 30th April and 18th July 2020, when there were no more responses on any of the forms. Only the principal investigator (PI) had access to the online data, which was downloaded and shared with the statisticians for analyses.

### Ethical consideration

The study was carried out according to the Declaration of Helsinki and the Checklist for Reporting Results of Internet E-Surveys [27]. As an institution requirement, we obtained ethical Institutional ethical approval from the Ethical Committee of the College of Basic and Applied Sciences at the University of Ghana (ECBAS 063/19-20). According to the global health ethics guidelines by the World Health Organization (WHO), studies, like this one, where there is no possibility of harm arising as a result of conducting the research can be exempted from ethical review. The survey was anonymous and participation was voluntary. Consent of participants was sought if they wanted to participate in the survey. Those who accepted were free to opt out if they changed their minds in the course of taking the survey, and such participants’ information was not recorded.

### Data analysis

Data from the online survey were extracted into Microsoft Excel for cleaning and verification. Descriptive statistics were used to describe demographics and research variables. Twelve factors assessing knowledge (29 items) were based on a true/false or yes/no basis and an additional “I don’t know”. A correct answer was assigned 1 point, and an incorrect/unknown answer was assigned 0 points. The total knowledge score ranged from 0 to 29. Participants’ overall knowledge was categorised (using Bloom’s cut-off point) as high if the score was between 80% to 100% (23–29 points); moderate if the score was between 60 to 79% (18–22.9 points), and low if the score was less than 60% (≤17 points). Bivariate analyses using Pearson’s Chi-square and Fisher’s exact tests were performed to examine the association of demographic factors with COVID-19 knowledge, attitude, and perception. Ordinal logistic regression was used to examine factors influencing COVID-19 knowledge. All significant variables (p-value < 0.25) from the bivariate analysis were included to estimate the final multivariable model. The goodness of fit was assessed using the Wald statistic. The adjusted odds ratio (OR) and 95% confidence intervals (CI) were computed, and the statistical significance level was set at *P <* 0.05. All analyses were done using Python statistical packages (Python Software Foundation. Python Language Reference, version 3.9 Available at http://www.python.org) and STATA software version 15.1 (StataCorp. LP, College Station, USA).

## Results

### Socio-demographic characteristics

A total of 1046 adults from 35 out of the targeted 48 SSA countries completed the questionnaire. The distribution of the respondents’ countries of origin and country of residence during the pandemic are presented in S3 and S4 Tables, respectively. Table 1 summarises participants’ socio-demographic characteristics. Participants median age was 33 years ranging from 18 to 76 years, and 50.5% were female. More than three-quarters (76.0%) were living in urban areas, and the mean household size was 4.9 ± 3.5. More than half of the participants were single (53.8%), including those who were never married, divorced, separated or widowed. About 43% of participants were either postgraduate or had completed their postgraduate studies, and the mean year of formal education was 18.1 ± 4.3. Four-fifths (80.9%) of the respondents were employed, either in the private or public sector, and 89.6% were Christians.

**Table 1:** Respondents’ socio-demographic characteristics by region of origin.

	Central Africa	East Africa	Southern Africa	West Africa	Total (1,046)
	(n = 293)	(n = 64)	(n = 53)	(n = 636)	
** Characteristics**	**n (%)**	**n (%)**	**n (%)**	**n (%)**	**n (%)**
**Age group (in years)**					
18–29	147 (50.2)	17 (26.5)	23 (43.4)	186 (29.2)	373 (35.7)
30–39	104 (35.5)	25 (39.1)	23 (43.4)	304 (47.8)	456 (43.6)
≥40	42 (14.3)	22 (34.4)	7 (13.2)	146 (23.0)	217 (20.7)
**Median Age (Range)**	29 (18–55)	33.5 (21–74)	30 (21–76)	34 (18–72)	33 (18–76)
**Gender**					
Male	166 (56.7)	34 (53.1)	22 (41.5)	306 (48.1)	528 (50.5)
Female	127 (43.3)	30 (46.9)	31 (58.5)	330 (51.9)	518 (49.5)
**Place of residence**					
Rural/Suburban	46 (15.7)	20 (31.3)	21 (39.6)	164 (25.8)	251 (24.0)
Urban	247 (84.3)	44 (68.7)	32 (60.4)	472 (74.2)	795 (76.0)
**Household size (Mean ± SD)**	4.4 ± 2.6	4.5 ± 2.5	4.5 ± 5.1	5.1 ± 3.8	4.9 ± 3.5
**Marital Status**					
Single*	202 (68.9)	26 (40.6)	47 (88.7)	288 (45.3)	563 (53.8)
Married	86 (29.4)	35 (54.7)	6 (11.3)	342 (53.8)	469 (44.8)
Prefer not to answer	5 (1.7)	3 (4.7)	0 (0.0)	6 (0.9)	14 (1.3)
**Educational level**					
Vocational/Secondary	37 (12.6)	2 (3.1)	9 (17.0)	29 (4.5)	77 (7.4)
Undergraduate	126 (43.0)	25 (39.1)	31 (58.5)	337 (53.0)	519 (49.6)
Postgraduate	130 (44.4)	37 (57.8)	13 (24.5)	270 (42.5)	450 (43.0)
**Total Years of Formal Education (Mean ± SD)**	17.7 ± 4.5	18.9 ± 3.8	16.6 ± 4.0	18.3 ± 4.3	18.1 ± 4.3
**Occupation**					
Employed	220 (75.1)	53 (82.8)	37 (69.8)	536 (84.3)	846 (80.9)
Student/Unemployed	73 (24.9)	11 (17.2)	16 (30.2)	100 (15.7)	200 (19.1)
**Religion**					
Christian	282 (96.2)	57 (89.1)	50 (94.3)	548 (86.2)	937 (89.6)
Islam	5 (1.7)	5 (7.8)	1 (1.9)	77 (17.1)	88 (8.4)
Others	6 (2.0)	2 (3.1)	2 (3.8)	11 (1.7)	21 (2.0)

*Single: Never married, divorced, separated, widow/widower.

### Respondents’ knowledge of COVID-19

**Fig 1:**
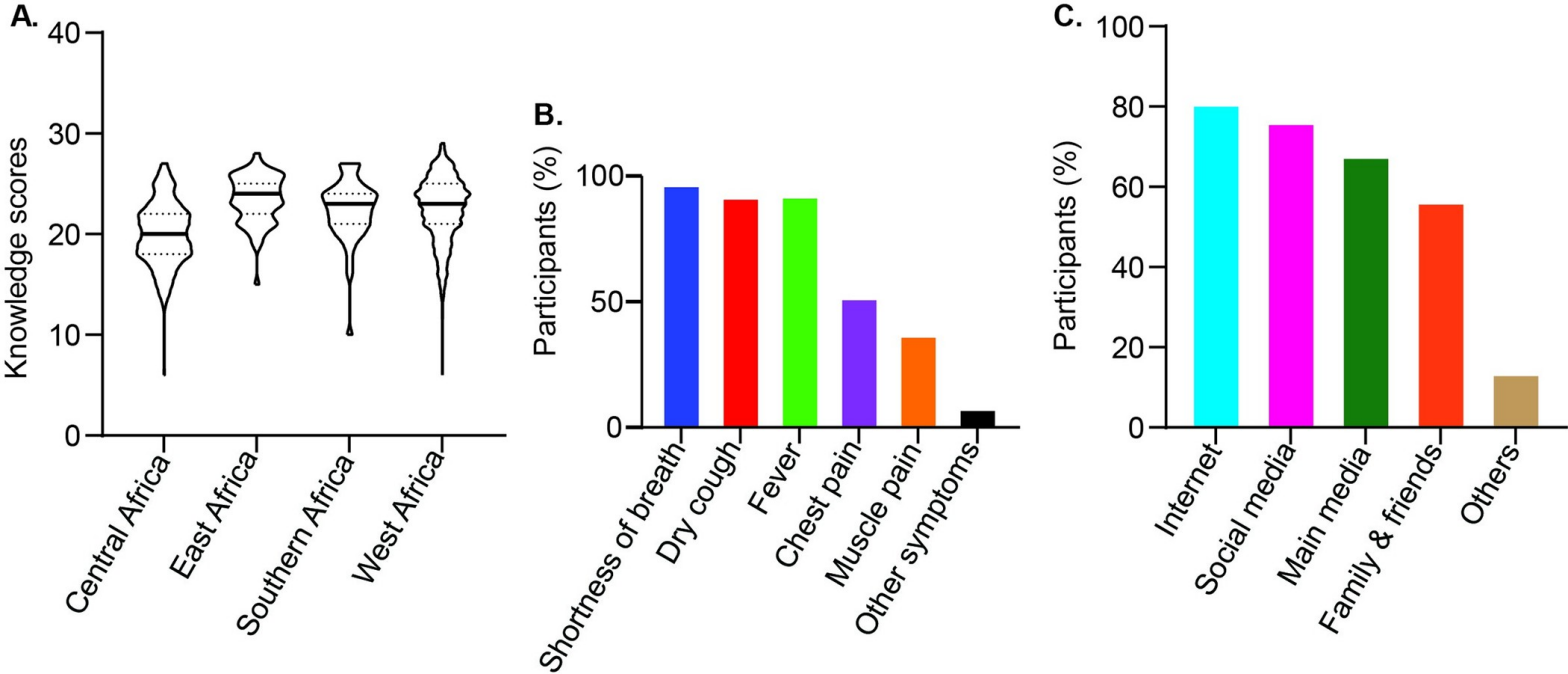
Respondents’ knowledge of COVID-19. **A**: Participants’ knowledge score, the horizontal lines across the violin plot represent the median knowledge scores whereas the dotted lines depict the 25^th^ and 75^th^ percentiles **B**: Participants identified COVID-19 main symptoms **C**: Participants Sources of COVID-19 information.

All participants had heard about COVID-19 mainly from the internet and social media (Fig 1C) but less than half (46.7%) had good knowledge of the disease (Table 2). Two-thirds of East Africans (68.8%) had a good COVID-19 knowledge score and one-fifth (21.2%) of Central Africans compared to other regions (P < 0.05); however, there was no significant difference between the regions (Fig 1A). Bivariate analysis showed significantly (p*<*0.05) associated with COVID-19 knowledge based on the region of origin, gender, educational level and occupation (Table 2). In the multivariate analyses, East (OR 7.9; 95% CI: 4.5–14; p *<* 0.001), Southern (OR 3.7; 95% CI: 2.1–6.5; p *<* 0.001) and West (OR 3.9; 95% CI: 2.9–5.2; p *<* 0.001) Africans had a good COVID-19 knowledge score than Central Africans (Table 2). Majority of the participants reported shortness of breath (96.7), dry cough (91.7%), and fever (92.2%) as the main COVID-19 symptoms (Fig 1B).

**Table 2:** Bivariate and multivariate analysis of factors influencing respondents’ knowledge score of COVID-19 (n = 1046).

	^a^Bivariate					^b^Multivariate	
	Low (n = 113)	Moderate (n = 445)	High (n = 488)				
	n (%)	n (%)	n (%)	Total	p-value	aOR(95%CI)	p-value
**Region of origin**							
Central Africa	57 (19.4)	174 (59.4)	62 (21.2)	293		1	
Southern Africa	3 (5.7)	22 (41.5)	28 (52.8)	53	<0.001	3.7 (2.1–6.5)	<0.001
West Africa	52 (8.2)	230 (36.2)	354 (55.6)	636		3.9 (2.9–5.2)	<0.001
East Africa	1 (1.5)	19 (29.7)	44 (68.8)	64		7.9 (4.5–14.0)	<0.001
**Age group (in years)**							
18–29	47 (12.6)	162 (43.4)	164 (44.0)	373			
30–39	48 (10.5)	191 (41.9)	217 (47.6)	456	0.475		
≥40	18 (8.3)	92 (42.4)	117 (49.3)	217			
**Gender**							
Male	65 (12.3)	235 (44.5)	228 (43.2)	528	0.051	1	
Female	48 (9.3)	210 (40.5)	260 (50.2)	518		1.2 (1.0–1.6)	0.083
**Place of residence**							
Rural/Suburban	20 (8.0)	107 (42.6)	124 (49.4)	251		1	
Urban	93 (11.7)	338 (42.5)	364 (45.8)	795	0.224	0.9 (0.8–1.3)	0.932
**Marital Status**							
Single	71 (12.6)	243 (43.2)	249 (44.2)	563		1	
Married	40 (8.5)	195 (41.6)	234 (49.9)	469	0.161	0.9 (0.7–1.2)	0.476
Prefer not to answer	2 (14.3)	7 (50.0)	5 (35.7)	14		0.6 (0.2–2.0)	0.426
**Educational level**							
Vocational/Secondary	8 (10.4)	47 (61.0)	22 (28.6)	77		1	
Undergraduate	48 (9.2)	218 (42.0)	253 (48.7)	519	0.004	1.2 (0.8–1.8)	0.447
Postgraduate	57 (12.7)	180 (40.0)	213 (47.3)	450		1.1 (0.7–1.7)	0.772
**Occupation**							
Employed	83 (9.8)	346(40.9)	417 (49.3)	846		1	
Student/Unemployed	30 (15.0)	99 (49.5)	71 (35.5)	200	0.001	0.6 (0.5–0.9)	0.007
**Religion**							
Christian	95 (10.1)	405(43.2)	437 (46.6)	937			
Islam	14 (15.9)	33 (37.5)	41 (46.6)	88	0.300		
Others	4 (19.0)	7 (33.3)	10 (47.6)	21			

^**a**^Fisher’s exact; aOR: Adjusted Odd Ratio; CI: Confidence interval

*Single (never married, divorced, separated, widow/widower).

**Fig 2:**
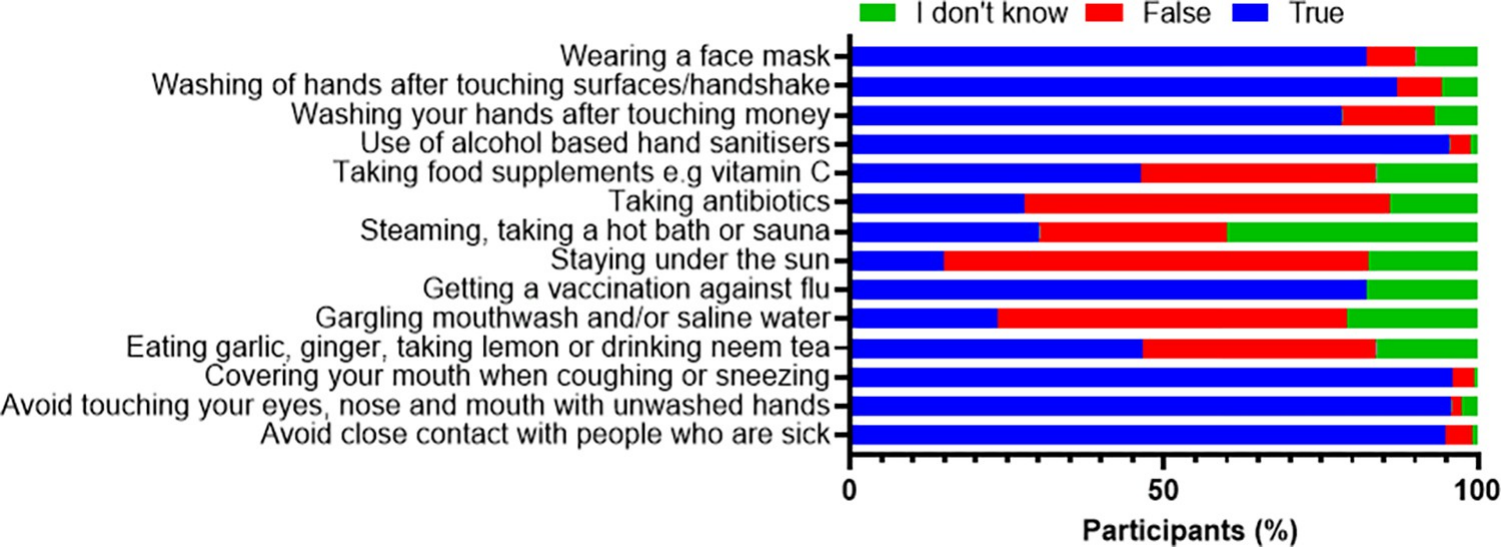
Responses on ways of preventing infection or spread of SARS-CoV-2. A chart showing the actions that respondents thought would help prevent getting infected with or spreading COVID-19. Respondents could pick multiple responses.

More than three-quarters (78.4% - 95.8%) of participants correctly identified standard measures that should be taken to avoid getting infected or spreading SARS-CoV-2 (Fig 2). Nearly half (46%) of the respondents believed also that taking garlic, ginger, lemon or neem tea and food supplements such as vitamin C, could prevent getting infected with the virus. Also, about 4 in 5 participants (82.2%) believe that getting a flu vaccine would prevent a person from getting infected with SARS-CoV-2. Likewise, taking antibiotics (27.7%), staying under the sun (14.9%) and steaming or taking a hot bath/sauna are also thought to prevent one from contracting the virus (Fig 2).

### Attitude of respondents towards COVID-19

**Fig 3:**
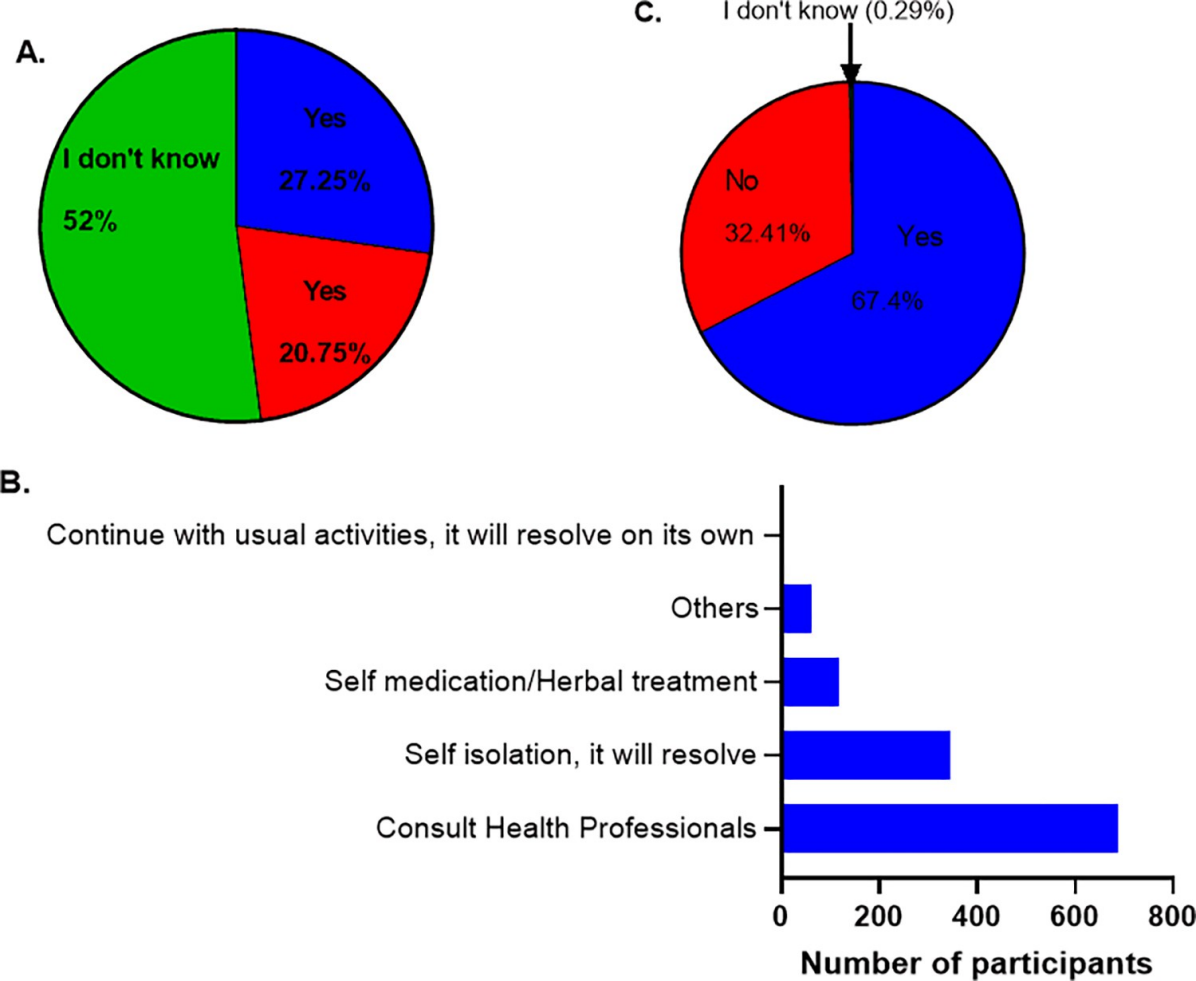
Attitude towards COVID-19 vaccine acceptance and SARS-CoV-2 infection. (**A)**: Respondents’ willingness to accept the COVID-19 vaccine. (**B):** Respondents’ attitude if they or their relatives contracted SARS-CoV-2. (**C):** Respondents’ level of concern towards contracting SARS-CoV-2.

Regarding COVID-19 vaccination, only 27.2% indicated willingness for vaccine acceptance while more than half (52%) were undecided (Fig 3A). Table 3 summarises the respondents’ attitudes towards a COVID-19 vaccine. The result showed significant association towards COVID-19 vaccine acceptance across different variables, including knowledge level (<0.001), region of origin (<0.001), age (0.006), gender, marital status and religion. Those with high knowledge of the disease were more likely to accept the COVID-19 vaccine (35.4%). Based on the region of origin, more than half of the participants from the East (54.7%) and about a third from Southern Africa (37.7%) would accept a COVID-19 vaccine compared to those from the West (28%) and Central (17.7%) regions. About 1 in 3 participants in the ≥40 age category was more willing to be vaccinated against SARS-CoV-2 infection than others, who were mostly undecided. Also, more males (31.3%) were willing to be vaccinated and nearly a third of undergraduates or those whose highest level of education was undergraduate level were more willing to take the SARS-CoV-2 vaccine.

**Table 3:** Analysis of factors influencing the willingness of respondents to accept the COVID-19 vaccine, if available (n = 1046).

	Yes (n = 285)	No (n = 217)	Undecided (544)	Total	
	n (%)	n (%)	n (%)	No.	*p-value*
**Knowledge score**					
Low	16 (14.2)	28 (24.8)	69 (61.0)	113	
Moderate	96 (21.6)	113 (25.4)	236 (53.0)	445	<0.001
High	173 (35.4)	76 (15.6)	239 (49.0)	488	
**Region of origin**					
Central Africa	52 (17.7)	98 (33.4)	143 (48.8)	293	
East Africa	35 (54.7)	18 (28.1)	11 (17.2)	64	<0.001
Southern Africa	20 (37.7)	12 (22.6)	21 (39.6)	53	
West Africa	178 (28.0)	89 (14.0)	369 (58.0)	636	
**Age group (In years)**					
18–29	110 (29.5)	69 (18.5)	194 (52.0)	373	
30–39	101 (22.1)	100 (21.9)	255 (55.9)	456	0.006
≥40	74 (34.1)	48 (22.1)	95 (43.8)	217	
**Gender**					
Male	165 (31.3)	87 (16.5)	276 (52.3)	528	
Female	120 (23.2)	130 (25.1)	268 (51.7)	518	<0.001
**Place of residence**					
Rural/Suburban	77 (30.7)	54 (21.5)	120 (47.8)	251	
Urban	208 (26.2)	163 (20.5)	424 (53.3)	795	0.267
**Marital Status** ^**a**^					
Single*	156 (27.7)	107 (19.0)	300 (53.3)	563	
Married	126 (26.9)	102 (21.7)	241 (51.4)	469	0.011
Prefer not to answer	3 (21.4)	8 (57.1)	3 (21.4)	14	
**Education level**					
Vocational/Secondary	21 (27.3)	18 (23.4)	38 (49.4)	77	
Undergraduate	151 (29.1)	103 (19.8)	265 (51.1)	519	
Postgraduate	113 (25.1)	96 (21.3)	241 (53.6)	450	0.674
**Occupation**					
Employed	232 (27.4)	185 (21.9)	429 (50.7)	846	
Student/Unemployed	53 (26.5)	32 (13.0)	115 (57.5)	200	0.124
**Religion**					
Christian	250 (26.7)	205 (21.9)	482 (51.4)	937	
Islam	27 (30.7)	7 (8.0)	54 (61.4)	88	
Others	8 (38.1)	5 (23.8)	8 (38.1)	21	0.023

^a^ Fisher’s exact

*Single (never married, divorced, separated, widow/widower).

With regards to action, if they or their relatives contracted SARS-CoV-2, 65.9% reported that they would seek medical attention from health professionals—going to the hospital or visiting a pharmacy (Fig 3B). Two in 3 respondents (67.4%) were worried about them or their relatives contracting the virus (Fig 3C).

### Perception of respondents on governments’ measures towards curbing SARS-CoV-2 spread

**Fig 4:**
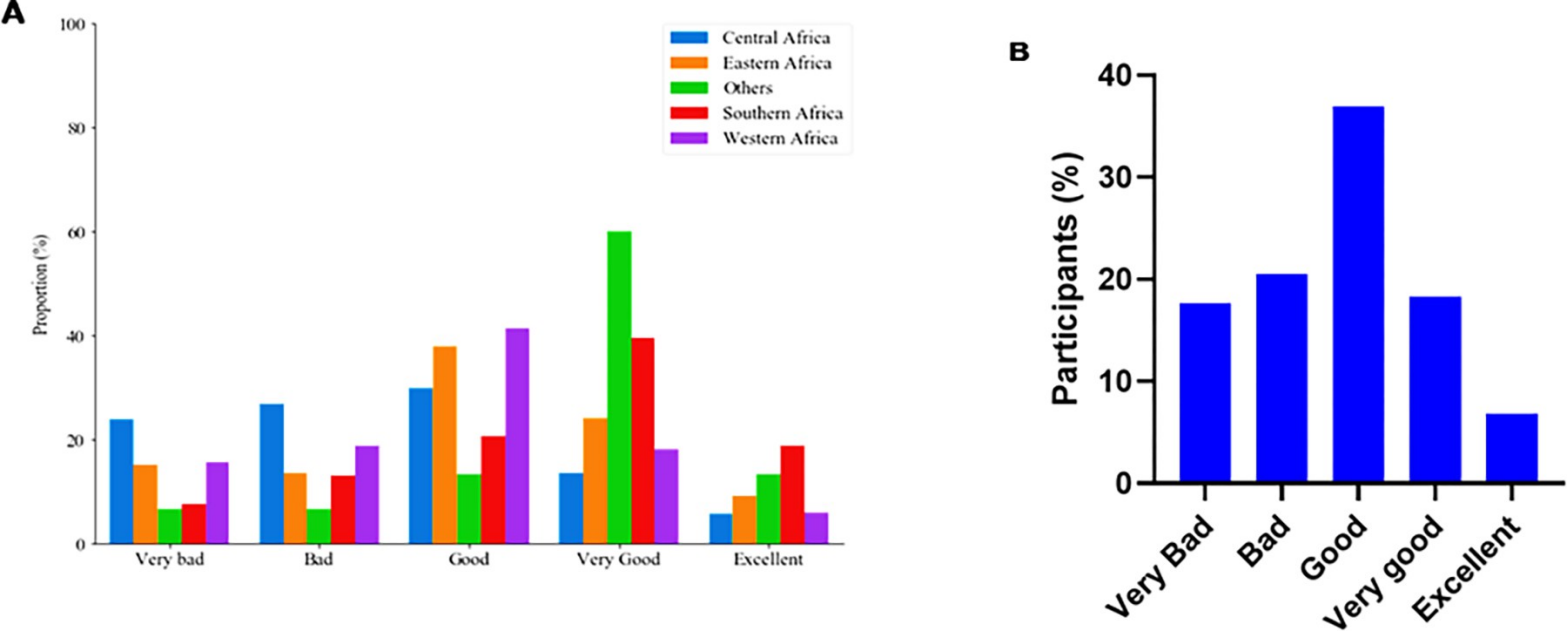
Perception of respondents on the measures employed by their governments to manage the COVID-19 pandemic. Respondents gave their perceptions on a scale ranging from bad to excellent. (**A)** Responses on how well governments managed the COVID-19 pandemic (according to region of residence). **(B)** General responses on government management of the pandemic.

Fig 4A shows the regional variation of the respondents’ perception regarding their governments’ response towards the COVID-19 pandemic while Fig 4B shows the general response of the study participants. On a scale of very bad to excellent, about 40% of the respondents from Southern, Western and East Africa agreed that the measures taken by their governments in the COVID-19 pandemic management was at least ‘good’ (Fig 4A). Generally, only 6.8% of the respondents perceived that their governments’ response towards curbing SARS-CoV-2 spread was ‘excellent’ while about a third (36%) felt that the response was ‘good’, and 17.6% felt that it was ‘very bad’ (Fig 4B). Table 4 shows participants’ perception of the government’s swiftness to curb the spread of SARS-CoV-2. Generally, more than one-third of respondents across all the socio-demographic categories agreed that their governments had taken prompt measures to curb the spread of the virus. The results further showed that level of knowledge on COVID-19, SSA region of residence, age and educational level were significantly associated (p<0.05) with the respondents’ perception towards their governments’ response to curbing the spread of COVID-19.

**Table 4:** Bivariate analysis of factors influencing respondents’ perception on measures taken to curb the spread of COVID-19 (n = 1046).

	Yes (n = 449)	No (n = 485)	Undecided (n = 112)		
	n (%)	n (%)	n (%)	Total	^a^p-value
**Knowledge level**					
Low	45 (39.8)	47 (41.6)	21 (18.6)	113	
Moderate	198 (44.5)	187 (42.0)	60 (13.5)	445	<0.001
High	206 (42.2)	251 (51.4)	31 (6.4)	488	
**Region of origin**					
Central Africa	102 (34.4)	151 (51.9)	40 (13.7)	291	
East Africa	41 (62.1)	21 (31.8)	4 (6.1)	66	<0.001^a^
Southern Africa	40 (75.5)	11 (20.8)	2 (3.8)	53	
West Africa	268 (42.1)	302 (47.5)	66 (10.4)	636	
**Age group (In years)**					
18–29	157 (42.1)	167 (44.8)	49 (13.1)	373	
30–39	182 (39.9)	225 (49.3)	49 (10.7)	456	0.022
≥40	110 (50.7)	93 (42.9)	14 (6.5)	217	
**Gender**					
Male	230 (43.6)	249 (47.2)	49 (9.3)	528	
Female	219 (42.3)	236 (45.6)	63 (12.2)	518	0.321
**Place of residence**					
Rural/Suburban	116 (46.2)	107 (42.6)	28 (11.2)	251	
Urban	333 (41.9)	378 (47.5)	84 (10.6)	795	0.389
**Marital Status**					
Single*	232 (41.2)	267 (47.4)	64 (11.4)	563	
Married	211 (45.0)	211 (45.0)	47 (10.0)	469	
Prefer not to answer	6 (42.9)	7 (50.0)	1 (7.1)	14	0.769^a^
**Education level**					
Vocational/Secondary	38 (49.4)	21 (27.3)	18 (23.4)	77	
Undergraduate	218 (42.0)	243 (46.8)	58 (11.2)	519	
Postgraduate	193 (42.9)	221 (49.1)	36 (8.0)	450	<0.001
**Occupation**					
Employed	355 (42.0)	403 (47.6)	88 (10.4)	846	
Student/Unemployed	94 (47.0)	82 (41.0)	24 (12.0)	200	0.237
**Religion**					
Christian	406 (43.3)	434 (46.3)	97 (10.4)	937	
Islam	33 (37.5)	42 (47.7)	13 (14.8)	88	0.675^a^
Others	10 (47.6)	9 (42.9)	2 (9.5)	21	

^a^ Fisher’s exact

*Single (never married, divorced, separated, widow/widower).

The responses were highly varied across the regions with the majority recording less than 50%.

#### Perception of participants on effective ways of handling COVID-19, where they run a greater risk of contracting SARS-CoV-2, and effective methods of reducing transmission

Majority of the respondents (>70%) across all socio-demographic categories agreed that isolation and treatment of COVID-19 patients were effective ways of handling COVID-19 (Table 5). The bivariate analysis showed that knowledge level, age, marital status, education level and occupation were significantly associated (p< 0.05) with the respondents’ perception on isolation and treatment of COVID-19 patients as effective ways of curbing the spread of the virus.

**Fig 5:**
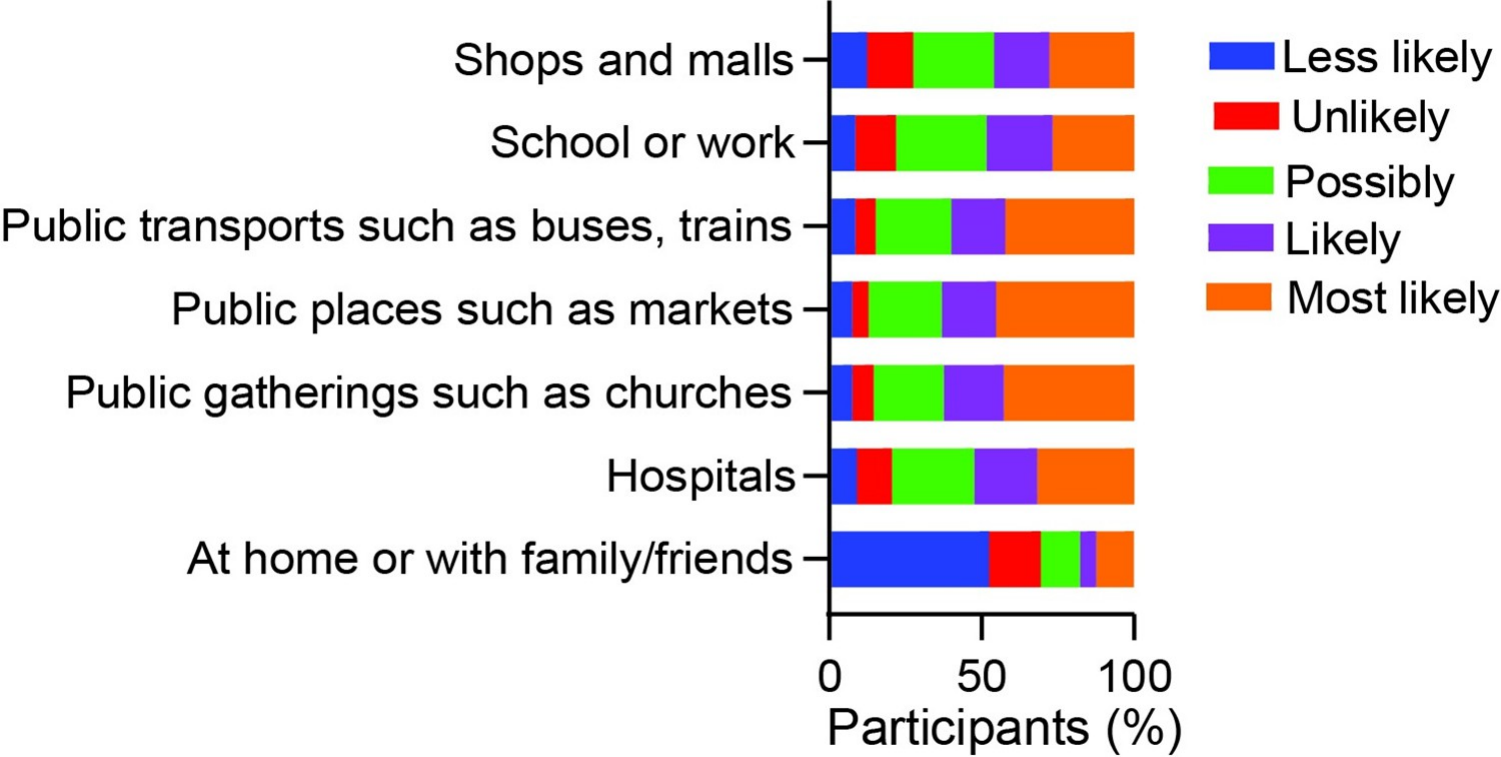
Respondents’ perception on where they are likely to contract SARS-CoV-2.

More than 40% of the respondents stated that they were most likely to contract SARS-CoV-2 at public places, including churches and markets and in public transports (Fig 5).

**Table 5:** Bivariate analysis of respondents’ perception on isolation and treatment of COVID-19 patients.

	Agree (n = 858)	Neutral (n = 33)	Disagree (n = 155)	Total	
	n (%)	n (%)	n (%)	No. (1046)	p-value
**Knowledge level**					
Low	95 (84.1)	9 (8.0)	9 (8.0)	113	
Moderate	361 (81.1)	15 (3.4)	69 (15.5)	445	0.004
High	402 (82.4)	9 (1.8)	77 (15.8)	488	
**Region of origin**					
Central Africa	240 (82.5)	15 (5.2)	36 (12.4)	291	
East Africa	52 (78.8)	3 (4.5)	11 (16.7)	66	
Southern Africa	47 (88.7)	0 (0.0)	6 (11.3)	53	0.132^a^
West Africa	519 (81.6)	15 (2.4)	102 (16.0)	636	
**Age group**					
18–29	308 (82.6)	22 (5.9)	43 (11.5)	376	
30–39	372 (81.6)	5 (1.1)	79 (17.3)	456	0.001
≥40	1780 (82.0)	6 (2.8)	33 (15.2)	217	
**Gender**					
Male	437 (82.8)	15 (2.8)	76 (14.4)	528	0.766
Female	421 (81.3)	18 (3.5)	79 (15.3)	518	
**Place of residence**					
Rural/Suburban	205 (81.7)	8 (3.2)	38 (15.1)	251	0.986
Urban	653 (82.1)	25 (3.1)	117 (14.7)	795	
**Marital Status**					
Single*	468 (83.1)	23 (4.1)	72 (12.8)	563	
Married	380 (81.0)	8 (1.7)	81 (17.3)	469	0.007^a^
Prefer not to answer	10 (71.4)	2 (14.3)	2 (14.3)	14	
**Education level**					
Vocational/Secondary	65 (84.4)	9 (11.7)	3 (3.9)	77	
Undergraduate	432 (83.2)	14 (2.7)	73 (14.1)	519	<0.001^a^
Postgraduate	361 (80.2)	10 (2.2)	79 (17.6)	450	
**Occupation**					
Employed	688 (81.9)	18 (2.1)	140 (16.5)	846	
Student/Unemployed	170 (85.0)	15 (7.5)	15 (7.5)	200	<0.001
**Religion**					
Christian	767 (81.9)	30 (3.2)	140 (14.9)	937	
Islam	73 (83.0)	3 (3.4)	12 (13.6)	88	0.934^a^
Others	18 (85.7)	0 (0.0)	3 (14.3)	21	

^a^ Fisher’s exact

*Single (never married, divorced, separated, widow/widower).

## Discussion

Pandemics and epidemics, when they occur, cause severe economic and health challenges on the affected nations/populations. Most epidemics just like COVID-19 are very contagious, with no known drugs. Therefore, one of the most effective means of effecting intervention strategies that will slow the rapid spread of the virus is assessing the populace knowledge, attitudes and perceptions of epidemics or pandemics soonest they occur. This is especially critical in the African context where several negative predictions were made [28]; hence, we conducted a knowledge, attitude and perception cross-sectional study among individuals of sub-Saharan African origin concerning the ongoing COVID-19 pandemic.

In our study, 89% of our respondents had average knowledge of COVID-19, with the Central African region having the least COVID-19 knowledge score compared to the other regions. The moderate COVID-19 knowledge is unexpected as most of our participants had a university first-degree, which would have inadvertently affected the knowledge reserve. This is comparable to what was obtained in Bangladesh and Ecuador [29, 30], but lower than that obtained in an Indonesian, China, or US study [31–33]. We also observed that our respondents were mostly internet surfers hugely biased towards social media platforms such as Facebook, Twitter, WhatsApp, etc. Social media misinformation can negatively impact mitigation against this pandemic as observed in a US study, where more than half of the respondents opined that not eating from a Chinese restaurant was an effective way of preventing SARS-CoV-2 transmission [31]. Since information on these various platforms is often uncertified and unverified [34], with false health information being circulated in most cases, it is imperative for governments and health organisations to utilise these platforms to disseminate accurate and reliable information. Our findings are similar to what was obtained in Nigeria [35], where more than half of the West African respondents’ originate.

Nevertheless, it is in contrast to a Chinese study, where an overwhelming 97% of the participants sourced their information from government websites [32], implying confidence of the population in government-sourced information. It is thus imperative for African governments to thrive on gaining such confidence of their citizens. This will encourage individuals to source reliable and valuable information from government sources [19, 32, 36].

Gender, region of origin, age, occupation, and education level were significant individual contributors to the respondents’ knowledge of the COVID-19 pandemic. Regionally, respondents from East, Southern and West Africa had appreciable COVID-19 knowledge; expectedly, having an associate degree, being a research scientist and/or a health professional further increased the respondents’ knowledge. A similar pattern has been observed in Ecuador, where having a higher educational level and increased age (older women), were significantly associated with COVID-19 optimistic behaviour on control strategies [30].

Most of our respondents knew SARS-CoV-2 preventive measures such as physical distancing, wearing nose masks, washing hands after touching money, avoiding touching the eyes, nose, or mouth without washing the hands. However, we did not evaluate whether this knowledge translated into practice. This is similar to other studies, where most participants were quite aware of SARS-CoV-2 preventive measures [29, 30, 32]. Another study found that while most of their respondents were quite knowledgeable about COVID-19 preventive measures, they did not necessarily practice them [37]. Therefore, there is a need to evaluate the practice of SARS-CoV-2 preventive measures in different African countries. Notedly, while almost half (45%) of our respondents opined that taking lemon, ginger, neem tea, or food supplements are useful preventive measures against SARS-CoV-2 infection, nearly one-fourth perceived that gargling mouthwash and/or saline water would play a similar preventive role. This information may have been obtained from numerous broadcast messages on different social media platforms, necessitating sharing verified information using different social media platforms.

Generally, more than half of our respondents (51.8%) were undecided about receiving a COVID-19 vaccine, while only about 27.6% are more receptive to being vaccinated against the disease. This shows that only about three in 10 people would accept to take a COVID-19 vaccine. Specifically, those with a first-degree, others who work in the formal sector, and East Africa participants, would accept a COVID-19 vaccine, if available. This is in contrast to a study carried out by the Africa Centres for Disease Control and Prevention (Africa CDC), in which they stated that at least 8 in 10 Africans would accept a COVID-19 vaccine, if available [38]. Interestingly, our results showed that participants from the Central African region were not willing to accept a COVID-19 vaccine and this could be due to their scepticism towards the way their government has handled the COVID-19 issue as observed in the negative responses towards government management of the pandemic. This once more highlights the importance of gaining the trust of the people in order to effectively disseminate public health information that would be readily accepted. Although attitudinal and behavioural acceptance of the COVID-19 vaccine seems to be rising across Europe, we are uncertain about the same results across SSA, with high variations across the different regions. In this study, respondents of East African origin (55.2%) showed more willingness to vaccine acceptance, which is similar to the Africa CDC study, where more Kenyans (64%) were more likely to accept the vaccine [38]. With the Democratic Republic of Congo still battling Ebola, it would be understandable why East Africans would have a positive attitude towards vaccination against SARS-CoV-2. However, vaccine acceptance among the large percentage of people who are undecided could be influenced by those who are unwilling to accept vaccination, probably due to safety concerns and infodemics, or by those willing to be vaccinated. This can go either way, seeing that COVID-19 vaccine acceptance has fallen in some Asian countries while rising in Europe [39].

The undecided behaviour towards immunization with COVID-19 vaccine could be combated by deliberate and concerted efforts by the ministries of health of various SSA countries to disseminate accurate information about the implications of accepting COVID-19 vaccine.

There seems to be a correlation between COVID-19 knowledge and vaccine acceptance as nearly two-thirds with low knowledge of the disease were undecided about accepting the vaccine. This shows a need for more public education on the role of vaccines in controlling infectious diseases and their safety as most people are afraid of the COVID-19 vaccine stemming from the precedent of the length of time it takes to develop a vaccine. The governments of SSA countries could also be more accountable and transparent to the citizens through the decision-making process, especially as regards this pandemic. This approach will ultimately increase citizens confidence and trust in the government.

Additionally, most of our respondents had a good perception of places that they could contract SARS-CoV-2, stating closed or crowded places, including churches, shopping malls, markets etc. as the most likely places to contract the virus. Furthermore, most respondents also opined that isolating and treating COVID-19 patients are effective ways of curtailing the virus’ spread. These positive perceptions could significantly mitigate disease spread. It is also unlike what was observed during the Ebola outbreak in 2014 where family members preferred to keep their sick relatives at home for fear of separation [40], thus leading to devasting effects in some of the affected countries. When the myth of isolation and treatment is broken through proper sensitisation, people become armed with the right information, and the desired results will be achieved in overcoming the COVID-19 pandemic.

The SARS-CoV-2 management was unique in Africa as most of our respondents alluded that their various governments undertook swift measures in curbing the spread of the infection. From 14th February 2020, when the first COVID-19 patient was identified in Egypt, the various African governments took swift and sometimes drastic measures to curb the virus spread, including border closure and restrictions in human gathering [41]. These efforts such as collectively setting up centralized laboratory hubs, coordinated by the Pathogen Genomics Intelligence Institute, or launching the Partnership to Accelerate COVID-19 Testing (PACT) towards enhancing health workers training across the continent to establishing a central procurement system at the Africa Centre for Disease and Control, and deploying one million community health workers for contact tracing [42] are laudable and should be strategically sustained.

## Conclusions

Although, the study methodology is limited by the high number of respondents from Nigeria and Cameroon, and biased towards internet users (being an online survey), and educated individuals, due to the network of the authors, these, however, does not preclude the findings of the study, which gives a broader perspective of SSAs’ knowledge, attitude and perception regarding the COVID-19 pandemic. The study will help to guide public health intervention in the subregion, tailoring measures to contribute towards the control of COVID-19 and possibly future similar events that may arise. Further studies may apply closed-ended questionnaires at the community level or phone interviews to target more groups of people who may not have internet access. With evolving new information on safety measures, emergent of new variants of concern, including increased transmission and immune evasion, controversial concerns about vaccine safety, public willingness to be vaccinated may significantly change, possibly from the time of data collection to publication. Utilizing diverse sources of information to sensitize the general population is required to get the correct information across to everyone. Also, ensuring internet access and media outlets, and increasing the level of interaction between governments, local agencies, and communities are necessary. Importantly, government information sources must be accurate, updated regularly, and widely disseminated. Finally, lessons from the effective control of the Ebola virus disease will be of utmost importance.

Further studies will be required to capture responses comparative to each SSA region populace to give a fairly complete picture of SSA population knowledge, attitude and perception concerning the COVID-19 pandemic. Although most respondents had a Bachelor’s degree and were urban dwellers, rural dwellers and people with little or no formal education were not fully captured in this study mostly due to limited or complete lack of internet access.

## Supporting information

S1 Table Sub-Saharan African countries targeted for the survey. (DOCX)

S2 Table List of survey questions according to category. (DOCX)

S3 Table Respondents’ countries of origin. (DOCX)

S4 Table Respondents’ country of residence during the pandemic. (DOCX)

S1 File The survey questionnaire in different languages. (A) English version of the survey questionnaire. (B) French version of the survey questionnaire. (C) Portuguese version of the survey questionnaire. (D) Spanish version of the survey questionnaire.
(DOCX)

S2 File Raw data for the study. (XLSX)
