# Supplementary material for: Knowledge, attitude and perception towards COVID-19 among representative educated sub-Saharan Africans: A cross-sectional study during the exponential phase of the pandemic
Source: PLoS One. 2024 Feb 1;19(2):e0281342. doi: 10.1371/journal.pone.0281342 (PMC10833576; doi:10.1371/journal.pone.0281342)
Supplement: S1 Table — (DOCX) [file pone.0281342.s001.docx]

**Knowledge, attitude and perception towards COVID-19 among representative educated sub-Saharan Africans: A cross-sectional study during the exponential phase of the pandemic**

Aniefiok John Udoakang^1*^, Nora Nghochuzie Nganyewo^1,2,3¶^, Alexandra Lindsey Djomkam Zune^1,2¶^, Charles Ochieng’ Olwal^1,2¶^, Nsikak-Abasi Aniefiok Etim^4¶^, Mary Aigbiremo Oboh^3^, Kesego Tapela^1,2^, Francis Dzabeng^1¶^, Samuel Mawuli Adadey^1,2^, Agnes Udoh^5^, Mazo Koné^6^, Joe Kimanthi Mutungi^1&*^, Peter Kojo Quashie^1,7,8&*^, Gordon Akanzuwine Awandare^1,2&^, Lily Paemka^1,2&*^

^1^ West African Centre for Cell Biology of Infectious Pathogens (WACCBIP), College of Basic and Applied Sciences, University of Ghana, Legon, Accra, Ghana

^2^ Department of Biochemistry, Cell and Molecular Biology, College of Basic and Applied Sciences, University of Ghana, Accra, Ghana

^3^ Medical Research Council Unit, The Gambia at the London School of Hygiene and Tropical Medicine, Banjul, The Gambia.

^4^ Department of Agricultural Economics and Extension, University of Uyo, Uyo, Akwa Ibom State, Nigeria

^5^ Jones school of Business, Rice University, Houston, Texas, USA

^6^ Department of Zoology, University of Ibadan, Ibadan, Oyo State, Nigeria

^7^ The Francis Crick Institute, London, United Kingdom

^8^ Virology Department, Noguchi Memorial Institute for Medical Research, University of Ghana, Legon, Accra, Ghana

*Corresponding authors

Email: [aniefiokjohn.udoakang@ucad.edu.sn](mailto:aniefiokjohn.udoakang@ucad.edu.sn); [mandith2004@yahoo.com](mailto:mandith2004@yahoo.com) (AJU)

[jkmutungi@ug.edu.gh](mailto:jkmutungi@ug.edu.gh); [joemutungi@gmail.com](mailto:joemutungi@gmail.com) (JKM)

[pquashie@ug.edu.gh](mailto:pquashie@ug.edu.gh) (PKQ); [leepaemka@gmail.com](mailto:leepaemka@gmail.com) (LP)

^¶^ These authors contributed equally to this work.

^&^ These authors also contributed equally to this work

**S1 Table: Sub-Saharan African countries targeted for the survey**

| **West Africa** | **East Africa** | **Central Africa** | **Southern Africa** |
| --- | --- | --- | --- |
| Benin | Comoros | Burundi | Angola |
| Burkina Faso | Djibouti | Cameroon | Botswana |
| Cape Verde | Eritrea | Central African Republic | Eswatini |
| Côte d’Ivoire | Ethiopia | Chad | Lesotho |
| The Gambia | Kenya | Congo Republic | Malawi |
| Ghana | Madagascar | Democratic Republic Congo | Mozambique |
| Guinea | Mauritius | Equatorial Guinea | Namibia |
| Guinea-Bissau | Rwanda | Gabon | South Africa |
| Liberia | Seychelles | São Tomé and Principe | Zambia |
| Mali | Somalia | South Sudan | Zimbabwe |
| Niger |  | Sudan |  |
| Nigeria |  | Tanzania |  |
| Senegal |  | Uganda |  |
| Sierra Leone |  |  |  |
| Togo |  |  |  |
