# Supplementary material for: Knowledge, attitude and perception towards COVID-19 among representative educated sub-Saharan Africans: A cross-sectional study during the exponential phase of the pandemic
Source: PLoS One. 2024 Feb 1;19(2):e0281342. doi: 10.1371/journal.pone.0281342 (PMC10833576; doi:10.1371/journal.pone.0281342)
Supplement: S2 Table — (DOCX) [file pone.0281342.s002.docx]

**Knowledge, attitude and perception towards COVID-19 among representative educated sub-Saharan Africans: A cross-sectional study during the exponential phase of the pandemic**

Aniefiok John Udoakang^1*^, Nora Nghochuzie Nganyewo^1,2,3¶^, Alexandra Lindsey Djomkam Zune^1,2¶^, Charles Ochieng’ Olwal^1,2¶^, Nsikak-Abasi Aniefiok Etim^4¶^, Mary Aigbiremo Oboh^3^, Kesego Tapela^1,2^, Francis Dzabeng^1¶^, Samuel Mawuli Adadey^1,2^, Agnes Udoh^5^, Mazo Koné^6^, Joe Kimanthi Mutungi^1&*^, Peter Kojo Quashie^1,7,8&*^, Gordon Akanzuwine Awandare^1,2&^, Lily Paemka^1,2&*^

^1^ West African Centre for Cell Biology of Infectious Pathogens (WACCBIP), College of Basic and Applied Sciences, University of Ghana, Legon, Accra, Ghana

^2^ Department of Biochemistry, Cell and Molecular Biology, College of Basic and Applied Sciences, University of Ghana, Accra, Ghana

^3^ Medical Research Council Unit, The Gambia at the London School of Hygiene and Tropical Medicine, Banjul, The Gambia.

^4^ Department of Agricultural Economics and Extension, University of Uyo, Uyo, Akwa Ibom State, Nigeria

^5^ Jones school of Business, Rice University, Houston, Texas, USA

^6^ Department of Zoology, University of Ibadan, Ibadan, Oyo State, Nigeria

^7^ The Francis Crick Institute, London, United Kingdom

^8^ Virology Department, Noguchi Memorial Institute for Medical Research, University of Ghana, Legon, Accra, Ghana

*Corresponding authors

Email: [aniefiokjohn.udoakang@ucad.edu.sn](mailto:aniefiokjohn.udoakang@ucad.edu.sn); [mandith2004@yahoo.com](mailto:mandith2004@yahoo.com) (AJU)

[jkmutungi@ug.edu.gh](mailto:jkmutungi@ug.edu.gh); [joemutungi@gmail.com](mailto:joemutungi@gmail.com) (JKM)

[pquashie@ug.edu.gh](mailto:pquashie@ug.edu.gh) (PKQ)

[leepaemka@gmail.com](mailto:leepaemka@gmail.com) (LP)

**Table S2: List of survey questions according to category**

| **Demographic** | **Knowledge** | **Attitude** | **Perception** |
| --- | --- | --- | --- |
| 1. What is your Nationality? * | 14. What is your source of information on COVID-19? Tick all that applies. * | 17. What will you do if you or a relative is diagnosed with COVID-19? * | 15. Are you worried that you or a close person may contract the virus? * |
| 2. What is your Nationality if you are not an African but reside on the continent. | 16. Have you or anybody that you know contacted COVID-19? * | 26. If there is a vaccine for COVID-19, would you accept to be vaccinated? * | 20. Where do you think you run a greater risk of contacting COVID-19? * |
| 3. What is your country of residence during this pandemic? * | 18. How is COVID-19 transmitted? (please tick all applicable) * |  | 21. On a scale of 1 - 5, 1 being very badly, how well do you think that your government is managing the spread of COVID-19? * |
| 4. Please, what is your country of residence if you do not live in Africa? | 19. Which of the following actions helps to prevent getting infected with or spreading COVID-19? * |  | 22. Do you think that prompt measures are being taken to curb the spread of the disease in your country? * |
| 5. Gender * | 23. The main clinical symptoms of COVID-19 are: |  | 29. Isolation and treatment of people who are infected with COVID-19 are effective ways to reduce the spread of the virus. * |
| 6. Age as at last birthday: * | 24. Is there currently a vaccine that protects against COVID-19? * |  |  |
| 7. Marital status * | 25. There is currently no effective cure for COVID-19, but early symptomatic and supportive treatment can help most patients recover from the infection. * |  |  |
| 8. How would you describe the area in which you live? * | 27. Unlike the common cold, stuffy nose, runny nose, and sneezing are less common in persons infected with the COVID-19. * |  |  |
| 9. How many people are living in your household including you? * | 28. Not all patients with COVID-19 will develop into severe cases. Only those who are elderly, have chronic illnesses, and are obese are more likely to be severe cases. * |  |  |
| 10. What is your religion? * | 30. Who should be tested for COVID-19 infection? |  |  |
| 11. What is your highest educational degree? * | 31. Who are the most at-risk people with COVID-19? |  |  |
| 12. Total number of years of formal education | 32. COVID-19 is diagnosed by isolating the virus from.... |  |  |
| 13. Occupation * |  |  |  |
|  |  |  |  |
