# Supplementary material for: Knowledge, attitude and perception towards COVID-19 among representative educated sub-Saharan Africans: A cross-sectional study during the exponential phase of the pandemic
Source: PLoS One. 2024 Feb 1;19(2):e0281342. doi: 10.1371/journal.pone.0281342 (PMC10833576; doi:10.1371/journal.pone.0281342)
Supplement: S1 File — (A) English version of the survey questionnaire. (B) French version of the survey questionnaire. (C) Portuguese version of the survey questionnaire. (D) Spanish version of the survey questionnaire. (DOCX) [file pone.0281342.s005.docx]

**Knowledge, attitude and perception towards COVID-19 among representative educated sub-Saharan Africans: A cross-sectional study during the exponential phase of the pandemic**

Aniefiok John Udoakang^1*^, Nora Nghochuzie Nganyewo^1,2,3¶^, Alexandra Lindsey Djomkam Zune^1,2¶^, Charles Ochieng’ Olwal^1,2¶^, Nsikak-Abasi Aniefiok Etim^4¶^, Mary Aigbiremo Oboh^3^, Kesego Tapela^1,2^, Francis Dzabeng^1¶^, Samuel Mawuli Adadey^1,2^, Agnes Udoh^5^, Mazo Koné^6^, Joe Kimanthi Mutungi^1&*^, Peter Kojo Quashie^1,7,8&*^, Gordon Akanzuwine Awandare^1,2&^, Lily Paemka^1,2&*^

^1^ West African Centre for Cell Biology of Infectious Pathogens (WACCBIP), College of Basic and Applied Sciences, University of Ghana, Legon, Accra, Ghana

^2^ Department of Biochemistry, Cell and Molecular Biology, College of Basic and Applied Sciences, University of Ghana, Accra, Ghana

^3^ Medical Research Council Unit, The Gambia at the London School of Hygiene and Tropical Medicine, Banjul, The Gambia.

^4^ Department of Agricultural Economics and Extension, University of Uyo, Uyo, Akwa Ibom State, Nigeria

^5^ Jones school of Business, Rice University, Houston, Texas, USA

^6^ Department of Zoology, University of Ibadan, Ibadan, Oyo State, Nigeria

^7^ The Francis Crick Institute, London, United Kingdom

^8^ Virology Department, Noguchi Memorial Institute for Medical Research, University of Ghana, Legon, Accra, Ghana

*Corresponding authors

Email: [aniefiokjohn.udoakang@ucad.edu.sn](mailto:aniefiokjohn.udoakang@ucad.edu.sn); [mandith2004@yahoo.com](mailto:mandith2004@yahoo.com) (AJU)

[jkmutungi@ug.edu.gh](mailto:jkmutungi@ug.edu.gh); [joemutungi@gmail.com](mailto:joemutungi@gmail.com) (JKM)

[pquashie@ug.edu.gh](mailto:pquashie@ug.edu.gh) (PKQ)

[leepaemka@gmail.com](mailto:leepaemka@gmail.com) (LP)

**Supplementary File: Questionnaire (Different versions)**

**A. English version**

**COVID-19: Knowledge, Attitude and Perception**

Dear participant,
We are researchers from the University of Ghana, Ghana; the University of Ibadan, Nigeria, and the Medical Research Council, Gambia, and are conducting a survey on the Knowledge, Attitude and Perception on COVID-19.
The outcome of this study will inform Stakeholders, Decision-makers, Scientists and the General public to take appropriate measures towards forecasting the spread of the virus as well as improve preparedness and response.
Your responses are treated anonymously and with the strictest confidentiality. The information will be used solely for research purposes, and this will take at least 5 minutes.
Thank you.

* Required

Do you accept to participate in this study? *

- Yes, I accept to participate in this study.
- No, I do not accept to participate in this study.

1. What is your Nationality? *

2. What is your nationality if you are not an African but reside on the continent.

3. What is your country of residence during this pandemic? *

4. Please, what is your country of residence if you do not live in Africa?

5. Gender *

6. Age as at last birthday: *

7. Marital status *

8. How would you describe the area in which you live? *

i. Urban

ii. Surburb

iii. Rural

9. How many people are living in your household, including you? *

10. What is your religion? *

11. What is your highest educational degree? *

12. Total number of years of formal education

13. Occupation *

14. What is your source of information on COVID-19? Tick all that applies. *

1. Internet websites
2. Social media (WhatsApp, Twitter, Instagram, Facebook, etc.)
3. Media (Television, Newspaper and Radio)
4. Family and Friends
5. Others:

15. Are you worried that you or a close person may contract the virus? *

16. Have you or anybody that you know contracted SARS-CoV-2? *

17. What will you do if you or a relative is diagnosed with COVID-19? *

1. Go to the hospital
2. Continue with usual activities, it will resolve on its own
3. Herbal treatment
4. Self-medication
5. Self-isolation, it will resolve on its own
6. Speak with a pharmacist
7. Others:

18. How is COVID-19 transmitted? (please tick all applicable) *

1. By respiratory droplets when an infected person coughs, sneezes or speaks.
2. Blood transfusion.
3. Contaminated foodstuffs.
4. Eating bushmeat or wild animals (like bat).
5. Handshake
6. Touching contaminated surfaces and then touching your face
7. I don’t know
8. Others:

19. Which of the following actions helps to prevent getting infected with or spreading COVID-19? *

1. Avoid close contact with people who are sick.
2. Avoid touching your eyes, nose and mouth with unwashed hands.
3. Covering your mouth when coughing or sneezing.
4. Eating garlic, ginger, taking lemon or drinking neem tea.
5. Gargling mouthwash and/or saline water.
6. Getting a vaccination against flu.
7. Staying under the sun.
8. Steaming, taking a hot bath or sauna
9. Taking antibiotics
10. Taking food supplements, e.g. vitamin C
11. Use of alcohol-based hand sanitisers
12. Washing your hands after touching money
13. Washing of hands after touching surfaces/handshake
14. Wearing a face mask

20. 1 being less likely and 5 being most likely, where do you think you run a greater risk of contacting COVID-19? *

1. At home or with family/friends
2. Hospitals
3. Public gatherings such as churches, mosques, cinemas and restaurants
4. Public places such as markets
5. Public transports such as buses, trains, airplanes, etc.
6. School or work
7. Shops and malls

21. On a scale of 1 - 5, 1 being very badly, how well do you think that your government is managing the spread of COVID-19? *

22. Do you think that prompt measures are being taken to curb the spread of the disease in your country? *

23. The main clinical symptoms of COVID-19 are (please tick all applicable) : *

1. Chest pain
2. Dry cough
3. Fever
4. Muscle pain
5. Shortness of breath
6. Other:

24. Is there currently a vaccine that protects against COVID-19? *

25. There is currently no effective cure for COVID-19, but early symptomatic and supportive treatment can help most patients recover from the infection. *

26. If there is a vaccine for COVID-19, would you accept to be vaccinated? *

27. Unlike the common cold, stuffy nose, runny nose, and sneezing are less common in persons infected with the COVID-19. *

28. Not all patients with COVID-19 will develop into severe cases. Only those who are elderly, have chronic illnesses, and are obese are more likely to be severe cases. *

29. Isolation and treatment of people who are infected with COVID-19 are effective ways to reduce the spread of the virus. *

1. Strongly disagree
2. Disagree
3. Neutral
4. Agree
5. Strongly agree

30. Who should be tested for COVID-19 infection? (please tick all applicable) *

1. Anyone who lives or works in a high risk setting eg. Health care workers, prisons or other closed settings
2. Anyone who is admitted into a hospital
3. People who have come in contact with any of the above
4. People who have recently returned from overseas
5. Randomly test people
6. Scientists whose research involves close contact with the virus or infected people
7. Other:

31. Who are the most at-risk people with COVID-19? Please, Tick all applicable *

1. Children
2. Elderly people
3. People in detention facilities, e.g. prisons
4. People in hostels and boarding facilities
5. People with compromised immune system
6. People with diagnosed chronic medical conditions
7. Youths

32. COVID-19 is diagnosed by isolating the virus from.... (please tick all applicable) *

1. Blood
2. Faecal sample
3. Fluid from the lungs
4. Sputum
5. Swabs from the back of the nose and throat
6. Urine
7. All of the above
8. I don’t know
9. Other:

33. It is natural to be tempted to look up the answer to a question. If you did, for approximately how many of the questions above did you look up the answer online or consult before responding? ……………………….

**B. French version**

**COVID-19: Connaissances, Attitudes et Perceptions en Afrique**

Cher participant,
Nous sommes des chercheurs de diverses universités en Afrique (Université Cheikh Anta Diop de Dakar, Senegal; Université du Ghana, Ghana; et Medical Research Council, Gambie). Nous menons une étude sur les connaissances, attitudes et perceptions sur COVID-19.
Les résultats de cette étude informeront les parties prenantes, les décideurs, les scientifiques et le grand public de prendre les mesures appropriées concernant cette pandémie. Toutes les réponses sont traitées de manière anonyme et avec la plus stricte confidentialité. Cela prendra au moins 7 minutes de votre temps.
Pour toute demande de renseignements, veuillez contacter le chercheur principal du Dr Udoakang au "[noraannlindsey@gmail.com](mailto:noraannlindsey@gmail.com)".
Je vous remercie.

* Required

Acceptez-vous de participer à cette étude?

1. Quelle est votre nationalité ? *

2. Veillez indiquer votre pays d'origine si vous n'êtes pas africain mais résidez sur le continent.

3. Quel est votre pays de résidence ? *

4. Veillez indiquer votre pays de résidence si vous ne résidez pas dans l'Afrique.

5. Sexe *

6. Âge au dernier anniversaire *

7. État civil *

8. Comment décririez-vous la région dans laquelle vous vivez ? *

i. Urbain

ii. Banlieue

iii. Rural

9. Combien de personnes vivent dans votre ménage, vous y compris ? *

10. Quelle est votre religion ? *

11. Quel est votre diplôme d'études le plus élevé ? *

12. Nombre d'années d'éducation formelle

13. Profession *

14. Quelle est votre source d'information concernant le COVID-19 ? Cochez toutes les réponses applicables. *

1. Internet
2. Réseaux sociaux (WhatsApp, Twitter, Instagram, Facebook, etc.)
3. Médias (télévision, journaux et radio)
4. Famille et Amis
5. Autres

15. Craignez-vous que vous ou l’un de vos proches puisse contracter le virus ? *

16. Est-ce que vous ou l'une de vos connaissances, a contacté COVID-19 ? *

17. Que ferez-vous si vous ou un membre de votre famille contactez COVID-19 ? *

1. Aller à l’hôpital
2. Poursuivre les activités habituelles, le virus disparaîtra
3. Traitement à base de plantes
4. Auto-médication
5. Auto-isolement, le virus disparaîtra
6. Consulter un pharmacien
7. Autres

18. Comment le COVID-19 est-il transmis ? (veuillez cocher toutes les cases applicables) *

1. Par des gouttelettes respiratoires lorsqu'une personne infectée tousse, éternue ou parle.
2. Transfusion sanguine
3. Nourriture contaminée
4. Manger de la viande de brousse ou des animaux sauvages (comme la chauve-souris)
5. Poignée de main
6. Toucher des surfaces contaminées et ensuite se toucher le visage
7. Je ne sais pas
8. Autres

19. Laquelle des actions suivantes contribue à prévenir l'infection ou la propagation du COVID-19 ? *

1. Éviter tout contact étroit avec les personnes malades
2. Éviter de se toucher les yeux, le nez et la bouche avec des mains non lavées
3. Se couvrir la bouche en toussant ou en éternuant
4. Manger de l'ail, du gingembre, prendre du citron ou boire un thé de neem
5. Bain de bouche et/ou eau salée pour se gargariser
6. Se faire vacciner contre la grippe
7. Rester sous le soleil
8. Prendre un bain de vapeur ou un sauna
9. Prendre des antibiotiques
10. Prendre des compléments alimentaires, par exemple de la vitamine C
11. Utilisation de désinfectants pour les mains à base d'alcool
12. Se laver les mains après avoir touché de l'argent
13. Lavage des mains après avoir touché des surfaces/saluer quelqu’un
14. Le port du masque

20. Sur une échelle de 1 à 5, où pensez-vous courir le plus grand risque de contacter COVID-19 ? 1 étant moins probable et 5 étant le plus probable. *

1. A la maison ou avec la famille/les amis
2. Hôpitaux
3. Les rassemblements publics tels que les églises, les mosquées, les cinémas et les restaurants
4. Les lieux publics tels que les marchés
5. Les transports publics tels que les bus, les trains, les avions, etc.
6. A l’école ou au travail
7. Boutiques et centres commerciaux

21. Sur une échelle de 1 à 5, 1 étant très mauvais, comment pensez-vous que votre gouvernement gère la propagation du COVID-19 ? *

22. Pensez-vous que des mesures rapides sont prises pour freiner la propagation de la maladie dans votre pays ? *

23 . Les principaux symptômes cliniques de COVID-19 sont (veuillez cocher toutes les cases applicables) : *

1. Douleurs thoraciques
2. Toux sèche
3. Fièvre
4. Douleurs musculaires
5. Essoufflement ou difficultés respiratoires
6. Autres

24. Existe-t-il actuellement un vaccin qui protège contre l'infection par le COVID-19 ? *

25. Il n'existe actuellement aucun remède efficace pour COVID-19, mais un traitement symptomatique précoce et de soutien pouvant aider la plupart des patients à se remettre de l'infection. *

26. S'il existe un vaccin pour COVID-19, accepteriez-vous d'être vacciné? *

27. Contrairement au rhume, la congestion nasale, l'écoulement nasal et les éternuements sont moins fréquents chez les personnes infectées par le COVID-19. *

28. Tous les cas de COVID-19 ne deviendront pas des cas graves. Seuls les personnes âgées, qui souffrent de maladies chroniques et qui sont obèses sont plus susceptibles d'être des cas graves. *

29. L'isolement et le traitement des personnes infectées par le COVID-19 sont des moyens efficaces pour réduire la propagation du virus. *

1. Fortement en déaccord
2. Désaccord
3. Neutre
4. En Accord
5. Fortement d'accord

30. Qui doit être testé pour l'infection à COVID-19 ? (veuillez cocher toutes les cases applicables) *

1. Toute personne qui vit ou travaille dans un environnement à haut risque, par exemple les travailleurs de la santé, les prisons ou d'autres environnements fermés
2. Toute personne admise dans un hôpital
3. Les personnes qui ont été en contact avec l'un des éléments ci-dessus
4. Personnes récemment revenues de l'étranger
5. Tester aléatoirement des personnes
6. Les scientifiques dont les recherches impliquent un contact étroit avec le virus ou des personnes infectées
7. Autres

31. Quelles sont les personnes les plus à risque de contacter le COVID-19 ? Veuillez cocher toutes les cases applicables *

1. Enfants
2. Personnes âgées
3. Les personnes dans les lieux de détention, par exemple les prisons
4. Les personnes en foyer et en internat
5. Personnes dont le système immunitaire est affaibli
6. Personnes souffrant de maladies chroniques diagnostiquées
7. Jeunes

32. Le COVID-19 est diagnostiqué en isolant le virus de (veuillez cocher toutes les cases applicables) *

1. Sang
2. Échantillon de selles
3. Fluides pulmonaires
4. Expectorations
5. Prélèvements nasal et de la gorge
6. Urine
7. Tout ce qui précède
8. Je ne sais pas
9. Autres

33. Il est naturel d'être tenté de chercher la réponse à une question. Pour combien des questions ci-dessus avez-vous cherché la réponse en ligne ou consulté avant de répondre ? *

**C. Portuguese Version**

**Top of Form**

**Novo coronavírus (Covid-19): conhecimento, atitude e percepção entre africanos**

Caro participante, somos pesquisadores de várias universidades da África. Estamos realizando um estudo sobre o conhecimento, atitude e percepção de indivíduos sobre questões relacionadas ao COVID-19 na África. O resultado deste estudo informará sobre o entendimento atual do Covid-19, que orientará os tomadores de decisão e as partes interessadas a tomar as medidas apropriadas. Por favor, forneça com precisão respostas às seguintes perguntas. Todas as respostas são tratadas de forma anônima e com a mais estrita confidencialidade. Isso levará no máximo 10 minutos. Obrigado pelo seu tempo. Para qualquer dúvida, entre em contato com [noraannlindsey@gmail.com](mailto:noraannlindsey@gmail.com).

* Required

1. Qual é o seu país de origem ? *

2. Por favor, indique seu país de origem se você não é um africano, mas reside no continente?

3. Qual é o seu país de residência? *

4. Por favor, indique seu país de residência se você não mora no continente.

5. Gênero *

6. Idade no último aniversário:

7. Estado civil *

8. Como você descreveria a área em que vive? *

1. Urbano
2. Subúrbio
3. Rural

9. Quantas pessoas vivem em sua casa, incluindo você? *

10. Qual é a sua religião?

11. Qual é o seu maior grau educacional? *

1. Primário
2. Junior Secondary
3. Secundário / Secundário
4. Grau de associado (qualquer diploma formal após o ensino médio, mas não bacharel)
5. diploma de bacharel
6. Mestrado
7. Grau profissional
8. Doutorado
9. Vocacional
10. Outras:

12. Número total de anos de educação formal

13. Ocupação *

14. Qual é a sua fonte de informação sobre o Covid-19? Marque tudo o que se aplica *

1. Sites da Internet
2. Mídias sociais (WhatsApp, Twitter, Instagram, Facebook, etc.)
3. Mídia (televisão, jornal e rádio)
4. Família e amigos

De outros:

15. Você está preocupado que você ou uma pessoa próxima possa contrair o vírus? *

16. Você ou alguém que você conhece entrou em contato com o Covid-19? *

17. O que você fará se você ou um parente for diagnosticado com Covid-19? *

1. Vá ao hospital
2. Tratamento à base de plantas
3. Automedicação
4. Auto-isolamento, ele resolverá por conta própria
5. Continue com as atividades habituais, ele resolverá por conta própria
6. De outros:

18. Como o Covid-19 é transmitido? (marque todas as opções aplicáveis) *

1. Por gotículas respiratórias quando uma pessoa infectada tosse, espirra ou fala.
2. Aperto de mão
3. Tocando superfícies contaminadas e depois tocando seu rosto
4. Transfusão de sangue
5. Alimentos contaminados
6. Comer carne de animais selvagens ou animais selvagens (como morcego)
7. Eu não sei
8. De outros:

19. Qual das seguintes ações ajuda a evitar a infecção ou a disseminação do Covid-19? *

1. Vestindo uma máscara facial
2. Lavando as mãos depois de tocar em dinheiro
3. Uso de desinfetantes para as mãos à base de álcool
4. Lavagem das mãos após tocar nas superfícies / aperto de mão
5. Comer alho, gengibre, tomar limão ou beber chá de nim
6. Como vacinar contra a gripe
7. Gargarejar enxaguatório bucal e / ou água salina
8. Evite tocar nos olhos, nariz e boca com as mãos não lavadas
9. Evite contato próximo com pessoas doentes
10. Tomar antibióticos
11. Cobrindo a boca ao tossir ou espirrar
12. Ficar sob o sol
13. Tomar suplementos alimentares, por exemplo, vitamina C
14. Vapor, tomar um banho quente ou sauna
15. Vestindo uma máscara facial
16. Lavando as mãos depois de tocar em dinheiro
17. Uso de desinfetantes para as mãos à base de álcool
18. Lavagem das mãos após tocar nas superfícies / aperto de mão
19. Comer alho, gengibre, tomar limão ou beber chá de nim
20. Como vacinar contra a gripe
21. Gargarejar enxaguatório bucal e / ou água salina
22. Evite tocar nos olhos, nariz e boca com as mãos não lavadas
23. Evite contato próximo com pessoas doentes
24. Tomar antibióticos
25. Cobrindo a boca ao tossir ou espirrar
26. Ficar sob o sol
27. Tomar suplementos alimentares, por exemplo, vitamina C
28. Vapor, tomar um banho quente ou sauna

20. Em uma escala de 1 a 5, onde você acha que corre um risco maior de entrar em contato com o Covid-19? 1 sendo menos provável e 5 sendo mais provável. *

1. Lugares públicos como mercados
2. Lojas e shoppings
3. Reuniões públicas, como igrejas, mesquitas, cinemas e restaurantes
4. Transportes públicos, como ônibus, trens, aviões, etc.
5. Hospitais
6. Escola ou trabalho
7. Em casa ou com a família / amigos
8. Lugares públicos como mercados
9. Lojas e shoppings
10. Reuniões públicas, como igrejas, mesquitas, cinemas e restaurantes
11. Transportes públicos, como ônibus, trens, aviões, etc.
12. Hospitais
13. Escola ou trabalho
14. Em casa ou com a família / amigos

21.Em uma escala de 1 a 5, sendo 1 muito ruim, como você acha que seu governo está administrando a disseminação do Covid-19?

22. Você acha que medidas imediatas estão sendo tomadas para conter a propagação da doença em seu país? *

23. Os principais sintomas clínicos do Covid-19 são (marque todos os aplicáveis ): *

1. Dor muscular
2. Tosse seca
3. Dor no peito
4. Febre
5. Falta de ar
6. Other:

24. Existe atualmente uma vacina que protege contra a infecção pelo Covid-19? *

25. Atualmente, não há cura eficaz para o COVID-2019, mas o tratamento sintomático e de suporte precoce pode ajudar a maioria dos pacientes a se recuperar da infecção. *

26. Se houver uma vacina para o Covid-19, você concorda em ser vacinado? *

27. Ao contrário do resfriado comum, nariz entupido, coriza e espirros são menos comuns em pessoas infectadas com o COVID-19. *

28. Nem todos os pacientes com infecções por Covid-19 evoluirão para casos graves. Somente aqueles que são idosos, têm doenças crônicas e são obesos têm maior probabilidade de serem casos graves. *

29. O isolamento e o tratamento de pessoas infectadas com COVID-19 são formas eficazes de reduzir a propagação do vírus. *

1. Discordo fortemente
2. Discordo
3. Neutro
4. Aceita
5. Concordo plenamente

30. Quem deve ser testado para a infecção por Covid-19? ( Por favor, marque todos aplicáveis) *

1. Quem vive ou trabalha em um ambiente de alto risco, por exemplo . Trabalhadores da saúde, prisões ou outros locais fechados
2. Cientistas cuja pesquisa envolve contato próximo com o vírus ou pessoas infectadas
3. Pessoas que retornaram recentemente do exterior
4. Pessoas que entraram em contato com qualquer um dos itens acima
5. Qualquer pessoa admitida em um hospital
6. Testar pessoas aleatoriamente
7. Other:

31. Quem são as pessoas mais em risco com o Covid-19? Marque todas as opções aplicáveis *

1. Pessoas com sistema imunológico comprometido
2. Pessoas com condições médicas crônicas diagnosticadas
3. Pessoas idosas
4. Crianças
5. Jovens
6. Pessoas em centros de detenção, por exemplo, prisões
7. Pessoas em albergues e instalações de embarque

32. Covid-19 é diagnosticada pelo isolamento do vírus a partir de .... ( por favor, marque todos aplicáveis) *

1. Cotonetes da parte de trás do nariz e da garganta
2. Fluido dos pulmões
3. Sangue
4. Escarro
5. Amostra fecal
6. Urina
7. Other:

33. É natural ser tentado a procurar a resposta para uma pergunta. Em caso afirmativo, por aproximadamente quantas das perguntas acima você consultou a resposta on-line ou consultou antes de responder?

Obrigado pelo seu tempo. Por favor, envie seu email para "[noraannlindsey@gmail.com](mailto:noraannlindsey@gmail.com)" se você deseja saber o resultado deste estudo.

Submit

**D. Spanish version**

**Top of Form**

**COVID-19: Conocimiento, Actitud y Percepción**

Estimado participante, somos investigadores de varias universidades de África. Estamos llevando a cabo un estudio sobre el conocimiento, la actitud y la percepción de las personas sobre cuestiones relacionadas con COVID-19 en África. El resultado de este estudio informará sobre la comprensión actual de Covid-19, que guiará a los tomadores de decisiones y las partes interesadas a tomar las medidas apropiadas. Amablemente proporcione con precisión las respuestas a las siguientes preguntas. Todas las respuestas se tratan de forma anónima y con la más estricta confidencialidad. Esto llevará como máximo 10 minutos. Gracias por tu tiempo. Para cualquier consulta, comuníquese con [noraannlindsey@gmail.com](mailto:noraannlindsey@gmail.com). Saludos cordiales *

¿Aceptas hacer la encuesta?

* Requerido

1. ¿Cuál es su país de origen?

2. Indique su país de origen si no es africano pero reside en el continente

3. ¿Cuál es su país de residencia? *

4. Por favor, ¿cuál es su país de residencia si no vive en África?

5. Género *

6. Edad como en el último cumpleaños *

7. Estado civil *

1. Soltero
2. Casado
3. Divorciado
4. Viuda
5. Viudo
6. Elige no responder

8. ¿Cómo describirías el área en la que vives? *

1. Ciudad / pueblo (urbano)
2. Ciudad / pueblo (suburbio)
3. Pueblo (rural)

9. ¿Cuántas personas viven en su hogar, incluido usted? *

10. ¿Cuál es tu religión? *

1. cristiano
2. islam
3. Tradicional
4. Ateo
5. Ninguna
6. Otros

11. ¿Cuál es tu título educativo más alto? *

1. Primario
2. Secundaria secundaria
3. Secundaria / secundaria superior
4. Grado asociado (cualquier título formal después de la escuela secundaria pero no una licenciatura)
5. licenciatura
6. Maestría
7. Titulo profesional
8. Doctorado
9. Profesional
10. Otros

12. Número total de años de educación formal

13. Ocupación *

14. ¿Cuál es su fuente de información sobre Covid-19? Marque todo lo que corresponda *

1. Sitios web de internet
2. Redes sociales ( WhatsApp , twitter, Instagram , Facebook, etc.)
3. Medios (Televisión, Periódicos y Radio)
4. Familiares y amigos
5. Otro

15. ¿Le preocupa que usted o una persona cercana pueda contraer el virus? *

16. ¿Usted o alguien que usted conoce ha contactado a Covid-19?

17. ¿Qué harás si tú o un pariente es diagnosticado con Covid-19? *

1. Ir al hospital
2. Tratamiento a base de hierbas
3. Automedicación
4. Hablar con un farmacéutico
5. Autoaislamiento , se resolverá por sí solo
6. Continúe con las actividades habituales, se resolverá por sí solo
7. Otro

18. ¿Cómo se transmite Covid-19? ( Por favor marque todas las aplicables) *

1. Por gotitas respiratorias cuando una persona infectada tose, estornuda o habla
2. Apretón de manos
3. Tocar superficies contaminadas y luego tocarse la cara
4. Transfusión de sangre
5. Alimentos contaminados
6. Comer carne de animales silvestres o animales salvajes (como murciélagos)
7. No lo sé
8. Otro

19. ¿Cuál de las siguientes acciones ayuda a evitar infectarse o propagar Covid-19? *

1. Usando una mascarilla
2. Lavarse las manos después de tocar dinero
3. Utilizar de manos a base de alcohol desinfectantes
4. Lavado de manos después de tocar superficies / apretón de manos
5. Comer ajo, jengibre, tomar limón o tomar té de neem
6. Vacunarse contra la gripe
7. Gárgaras enjuague bucal y / o agua salina
8. Evite tocarse los ojos, la nariz y la boca con las manos sin lavar
9. Evitar el contacto cercano con personas que están enfermas
10. Tomar antibióticos
11. Cubrirse la boca al toser o estornudar
12. Permanecer bajo el sol
13. Tomar suplementos alimenticios, por ejemplo, vitamina C
14. Vapor, tomar un baño caliente o sauna
15. Usando una mascarilla
16. Lavarse las manos después de tocar dinero
17. Utilizar de manos a base de alcohol desinfectantes
18. Lavado de manos después de tocar superficies / apretón de manos
19. Comer ajo, jengibre, tomar limón o tomar té de neem
20. Vacunarse contra la gripe
21. Gárgaras enjuague bucal y / o agua salina
22. Evite tocarse los ojos, la nariz y la boca con las manos sin lavar
23. Evitar el contacto cercano con personas que están enfermas
24. Tomar antibióticos
25. Cubrirse la boca al toser o estornudar
26. Permanecer bajo el sol
27. Tomar suplementos alimenticios, por ejemplo, vitamina C
28. Vapor, tomar un baño caliente o sauna
29. 20. En una escala de 1-5, ¿dónde crees que corres un mayor riesgo de contactar a Covid-19? 1 es menos probable y 5 es más probable *
30. Lugares públicos como mercados
31. Tiendas y centros comerciales
32. Reuniones públicas como iglesias, mezquitas, cines y restaurantes
33. Transportes públicos como autobuses, trenes, aviones, etc
34. Hospitales
35. Escuela o trabajo
36. En casa o con familiares / amigos
37. Lugares públicos como mercados
38. Tiendas y centros comerciales
39. Reuniones públicas como iglesias, mezquitas, cines y restaurantes
40. Transportes públicos como autobuses, trenes, aviones, etc
41. Hospitales
42. Escuela o trabajo
43. En casa o con familiares / amigos

21. En una escala de 1 a 5, siendo 1 muy mal, ¿qué tan bien crees que tu gobierno está manejando la propagación de Covid-19? *

22. ¿Cree que se están tomando medidas rápidas para frenar la propagación de la enfermedad en su país? *

23. Los principales síntomas clínicos de Covid-19 son (marque todos los que correspondan): *

1. Dolor muscular
2. Tos seca
3. Dolor en el pecho
4. Fiebre
5. Dificultad para respirar
6. Otro

24. ¿Existe actualmente una vacuna disponible que proteja contra la infección con Covid-19? *

25. Actualmente no existe una cura efectiva para COVID-2019, pero el tratamiento sintomático y de apoyo temprano puede ayudar a la mayoría de los pacientes a recuperarse de la infección. *

26. Si hay una vacuna para Covid-19, ¿aceptaría vacunarse? *

27. A diferencia del resfriado común, la congestión nasal, la secreción nasal y los estornudos son menos comunes en personas infectadas con el COVID-19. *

28. No todos los pacientes con infecciones por Covid-19 se desarrollarán en casos graves. Solo aquellos que son ancianos, tienen enfermedades crónicas y son obesos tienen más probabilidades de ser casos graves *

29. El aislamiento y el tratamiento de personas infectadas con COVID-19 son formas efectivas de reducir la propagación del virus. *

1. Muy en desacuerdo
2. Discrepar
3. Neutral
4. De acuerdo
5. Totalmente de acuerdo

30. ¿Quién debe hacerse la prueba de infección por Covid-19? ( Por favor marque todas las aplicables) *

1. Cualquier persona que vive o trabaja en un entorno de alto riesgo, por ejemplo . Trabajadores de la salud, cárceles u otros entornos cerrados
2. Científicos cuya investigación involucra contacto cercano con el virus o personas infectadas
3. Personas que han regresado recientemente del extranjero
4. Personas que han entrado en contacto con cualquiera de los anteriores
5. Cualquier persona que ingrese en un hospital
6. Prueba al azar de personas
7. Otro

31. ¿Quiénes son las personas con mayor riesgo con Covid-19? Por favor, marque todos los aplicables *

1. Personas con sistema inmunitario comprometido
2. Personas con afecciones médicas crónicas diagnosticadas
3. Personas de edad avanzada
4. Niños
5. Jóvenes
6. Personas en centros de detención, por ejemplo, cárceles
7. Personas en albergues y pensiones

32. Covid-19 se diagnostica mediante el aislamiento del virus a partir de .... ( por favor marque todas las aplicables) *

1. Hisopos de la parte posterior de la nariz y la garganta
2. Fluido de los pulmones
3. Sangre
4. Esputo
5. Muestra fecal
6. Orina
7. Otro

33. Es natural sentirse tentado a buscar la respuesta a una pregunta. Si lo hizo, ¿aproximadamente cuántas de las preguntas anteriores buscó la respuesta en línea o la consultó antes de responder?

Gracias por tu tiempo. Envíe su correo electrónico a "[noraannlindsey@gmail.com](mailto:noraannlindsey@gmail.com)" si desea conocer el resultado de este estudio

Submit
